# LncRNADisease 2.0: an updated database of long non-coding RNA-associated diseases

**DOI:** 10.1093/nar/gky905

**Published:** 2018-10-04

**Authors:** Zhenyu Bao, Zhen Yang, Zhou Huang, Yiran Zhou, Qinghua Cui, Dong Dong

**Affiliations:** 1 Shanghai Key Laboratory of Regulatory Biology, Institute of Biomedical Sciences, School of Life Sciences, East China Normal University, Shanghai 200241, China; 2 Department of Biomedical Informatics, School of Basic Medical Sciences, MOE Key Lab of Cardiovascular Sciences, Center for Noncoding RNA Medicine, Peking University, Beijing 100190, China; 3 Institute of Biomedical Sciences, Fudan University, Shanghai, 200032, China; 4 Center of Bioinformatics, Key Laboratory for Neuro-Information of Ministry of Education, School of Life Science and Technology, University of Electronic Science and Technology of China, Chengdu, 610054, China

## Abstract

Mounting evidence suggested that dysfunction of long non-coding RNAs (lncRNAs) is involved in a wide variety of diseases. A knowledgebase with systematic collection and curation of lncRNA-disease associations is critically important for further examining their underlying molecular mechanisms. In 2013, we presented the first release of LncRNADisease, representing a database for collection of experimental supported lncRNA-disease associations. Here, we describe an update of the database. The new developments in LncRNADisease 2.0 include (i) an over 40-fold lncRNA-disease association enhancement compared with the previous version; (ii) providing the transcriptional regulatory relationships among lncRNA, mRNA and miRNA; (iii) providing a confidence score for each lncRNA-disease association; (iv) integrating experimentally supported circular RNA disease associations. LncRNADisease 2.0 documents more than 200 000 lncRNA-disease associations. We expect that this database will continue to serve as a valuable source for potential clinical application related to lncRNAs. LncRNADisease 2.0 is freely available at http://www.rnanut.net/lncrnadisease/.

## INTRODUCTION

Large number of studies have indicated that long non-coding RNAs (lncRNAs, >200 nt in length) are highly associated with the progression of a wide variety of diseases (1,2). Over the past decade, associations between dysfunction of lncRNAs and diseases have been the subject of intense investigation (3). A tremendous amount of experimentally and/or computationally supported lncRNA-disease associations have been identified (4,5). These disease-related lncRNAs offer potential new clinical application.

Previously, we developed LncRNADisease database (6), which integrated experimentally supported lncRNA-disease associations. The first version of LncRNADisease provided users an easy to use resource and platform to retrieve disease-related lncRNAs. Since its first release in 2013, more lncRNA-disease associations have been identified based on experimental and/or computational methods (5,7,8). It is therefore paramount to update LncRNADisease database to keep a pace with the rate of data accrual. Here, we introduce LncRNADisease 2.0, a significantly expanded version of this database. LncRNADisease 2.0 offers several distinct advantages from its first release: (i) integration from experimentally and/or computationally supported data, exceeding an over 40-fold lncRNA-disease associations enhancement over the previous version; (ii) providing the transcriptional regulatory relationships among lncRNA, mRNA and miRNA; (iii) mapping disease names to disease ontology (DO) (9) and Medical Subject Headings (MeSH) (10); (iv) providing a confidence score for each lncRNA-disease association. In addition, circular RNAs (circRNAs), a class of long endogenous non-coding RNAs (>100 nt in length), are associated with a wide range of diseases (11,12). CircRNA has also been discovered as an important type of lncRNA (13). We therefore integrated experimentally supported circRNA-disease associations into LncRNADisease 2.0 through manual literature curation. The database can be freely available at http://www.rnanut.net/lncrnadisease/.

## DATA COLLECTION AND DATABASE CONTENT

LncRNADisease 2.0 contains experimentally and/or computationally supported data. For experimentally supported data, we searched the PubMed (before 31 May 2018) using the following keywords: ‘long non-coding RNA’, ‘lncRNA’, ‘lincRNA’, ‘circular RNA’ and ‘circRNA’, in combination with ‘disease’, ‘cancer’ and ‘tumor’. Then, we retrieved the entries that describe the associations between lncRNAs/circRNAs and diseases from these publications. All selected literatures were manually curated by at least two researchers. More than 12 000 published literatures were curated, and 3878 literatures were recovered for lncRNA/circRNA-disease associations. We then separated those literatures covered in previous databases, (LncRNADisease (6), Lnc2Cancer (14), lncRNAdb (15) and NSDNA (16)) from newly identified ones, which led to 1416 new publications. For computationally supported data, the results predicted based on four algorithms (LRLSLDA (17), LDAP (18), RWRlncD (19) and LncDisease (20)) were derived. After mapping the overlap between experimentally and computationally supported data, 76% of experimentally supported data can be computationally predicted, suggesting the specificity and efficacy of computationally predicted data.

In LncRNADisease 2.0, the lncRNA-disease associations were curated from different types of resources under one common framework, including experimental and computational prediction evidence. Similar to miRTarBase database, the experimental evidence was divided into strong experimental evidence (e.g. qRT-PCR and northern blot) and weak experimental evidence (e.g. microarray and RNA sequencing) by a manual assignment. RNA-seq and microarray are high-throughput procedure for global gene expression profiling which may not exactly reflect the expression status of all gene. These diverse evidence contribute unequally to the identification of a specific lncRNA-disease association. A confidence score system was developed to evaluate the reliability of a specific lncRNA-disease association by integrating different evidence resources (21).

In principle, we assume that (i) experimentally supported evidence contributes more significantly to the confidence score than computationally supported evidences; (ii) strong experimental evidence are considered to be more reliable than weak experimental evidence; and (iii) the entries supported by more evidence should have significantly higher confidence scores than those supported by one evidence. Therefore, the confidence score depends on the evidence types and the number of evidence resources. The probability disjunction formula has been widely employed to measure combined scores in the case that multiple pieces of evidence exist (22,23). The confidence scores (CS) can be calculated as follows:

\begin{equation*}{\rm Confidence}\ {\rm Score}\ \ \left( {{\rm CS}} \right) = \ 1 - \mathop \prod \limits_t \left( {1 - \frac{{{w_t}}}{{1 + {e^{ - n}}}}} \right)\end{equation*}

where *t* is the evidence type (*s*: strong experimental evidence, *w*: weak experimental evidence, *p*: computational evidence), *n* is the number of evidence and *w_t_* is the weight factor. We assumed that lncRNA-disease associations supported by more computational evidence should not be given higher confidence scores than those supported by experimental evidence. So, *w_s_, w_w_* and *w_p_* is set to 1, 0.75 and 0.25, respectively. The confidence scores of well-supported lncRNA-disease associations are close to 1.

It is well known that many lncRNAs function together with microRNA and mRNA by forming a well-regulated interacting network (24). Here, we attempted to propose the potential relationships between lncRNAs, microRNAs and mRNAs. We defined the lncRNA–mRNA interactions by measuring the *cis*-regulatory function of lncRNAs (defined as pairs of lncRNA and mRNA located within a genomic window of 100 kb). MicroRNA was derived from miRBase (25), and microRNA targets were predicted using PITA (26), miRanda (27) and RNAhybrid (28) algorithms, and a high-quality miRNA target dataset was generated by intersecting data generated by at least two different miRNA target prediction methods. At last, LncRNADisease 2.0 contains 12207 lncRNA–mRNA and 2368 miRNA–lncRNA regulatory relationships.

**Figure 1.**
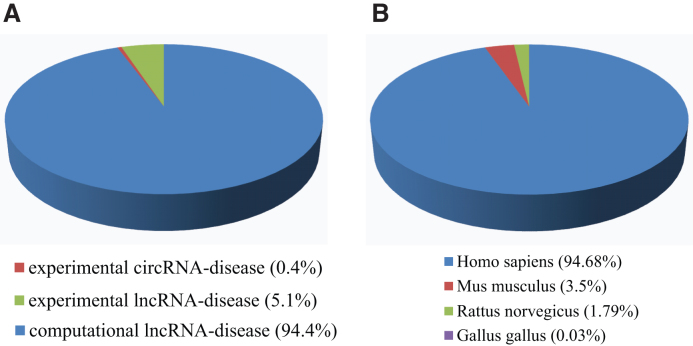
Statistics of diverse lncRNA-disease associations. (**A**) The percentage of diverse lncRNA-disease associations in LncRNADisease 2.0 database; (**B**) The percentage of lncRNA-disease associations across different species.

We manually curated 19 166 lncRNAs, 823 circRNAs and 529 diseases from 3878 literatures. Cancer (44.2%), cardiovascular disease (11.6%) and neurodegeneration disease (7.3%) represent the top three classes of diseases. LncRNADisease 2.0 contains 10 564 experimentally supported lncRNA-disease associations and 1004 experimentally supported circRNA-disease associations across four species (Figure 1). A total of 195 395 predicted lncRNA-disease associations were involved in our database, and 23102 entries can be predicted by at least two algorithms.

## DATABASE CONSTRUCTION

A user-friendly web interface was developed to present LncRNADisease 2.0. All data were managed by a relational database implemented with MySQL. The web interface for browsing and searching was implemented by PHP and JavaScript program. Apache Tomcat web server was used for the http server.

## NEW FEATURES AND DATABASE UTILITY

### Expanded entries on lncRNA-disease associations

Recent experimental technologies and computational prediction algorithms have been developed, leading to the expansion of many diverse lncRNA-disease associations. In LncRNADisease 2.0, an over 40-fold increase in lncRNA-disease association enhancement were obtained compared with the previous version (Table 1). We compared the content of LncRNADisease 2.0 with other related database (Table 1), including lncRNAdb (15), Lnc2Cancer (14), EVLncRNAs (29), which are now still available to download. After comparison to related database, the result showed that all these data were involved in LncRNADisease 2.0, and our database will be an important complement to other similar resources. In this release, experimentally supported circRNA-disease associations were also involved. Notably, we assigned confidence score to each entry by integrating the experimental and computational evidence.

**Table 1.** Data summary in LncRNADisease database

Data content	Version 2.0	Version 1.0	lncRNAdb	Lnc2Cancer	EVLncRNAs
lncRNA genes	19 166	321	71	531	4502
circRNA genes	823	NA	NA	NA	NA
Diseases	529	166	NA	86	338
Experimental lncRNA-disease associations	10 564	480	NA	1057	2324
Computational lncRNA-disease associations	195 395	1564	NA	NA	NA
circRNA-disease associations	1004	NA	NA	NA	NA
Interactions	14 575	475	307	NA	1163

### Database query and search platform

A user-friendly web interface was developed to present the LncRNADisease 2.0. Users can browse and search all lncRNA-disease associations in the database. LncRNADisease 2.0 also provided an option in the ‘Search’ page that allows users to filter lncRNA-disease associations by certain experimental methods. In LncRNADisease 2.0, each lncRNA-disease association entry contains detailed information, including gene symbol, gene category, disease information, regulatory relationship, PubMed information, etc. To facilitate users accessing disease information from external resources, the disease names were mapped to the DO and MeSH.

### LncRNA regulatory network

LncRNAs play increasingly appreciated gene-regulatory roles, and can affect an abundant number of target genes by interacting with sponging miRNAs. To further annotation the functional implication of disease-related lncRNAs, we constructed and visualized lncRNA–miRNA–mRNA network in LncRNADisease 2.0. It was developed on the basis of Cytoscape web program.

## CONCLUSION

Emerging evidence suggests that deregulation of lncRNAs plays an important role in diseases. To date, substantial studies have documented numerous lncRNAs involved in the progression of pathological disorders. Most disease-related lncRNAs have been examined in independent studies, and lncRNA-disease associations are scattered in various resources. Comprehensive collection of these lncRNA-disease associations will provide scientists with a resource for disease research. Here, we provided the LncRNADisease 2.0 to curate these data and provided a platform to facilitate the study of lncRNA-disease associations. LncRNADisease 2.0 contains more lncRNA-disease associations. We expect that the number of disease-related lncRNAs will continue to increase in the future release. We will continually maintain and update LncRNADisease database and integrate more related datasets, such as genomic and epigenetic information. We expect that this database will continue to serve as a valuable source for potential clinical application related to lncRNAs.
